# Low Adherence to the Mediterranean Diet Is Associated with Poor Socioeconomic Status and Younger Age: A Cross-Sectional Analysis of the EpiDoC Cohort

**DOI:** 10.3390/nu14061239

**Published:** 2022-03-15

**Authors:** Nuno Mendonça, Maria João Gregório, Clara Salvador, Ana Rita Henriques, Helena Canhão, Ana M. Rodrigues

**Affiliations:** 1 Comprehensive Health Research Centre (CHRC), NOVA Medical School, Universidade Nova de Lisboa (NMS/UNL), 1099-085 Lisboa, Portugal; nuno.mendonca@nms.unl.pt (N.M.); mariajoaobg@gmail.com (M.J.G.); salvadorclf@gmail.com (C.S.); anarita.henriques@nms.unl.pt (A.R.H.); helena.canhao@nms.unl.pt (H.C.); 2 EpiDoC Unit, Centro de Estudos de Doenças Crónicas (CEDOC), NOVA Medical School, Universidade Nova de Lisboa (NMS/UNL), 1150-082 Lisboa, Portugal; 3 EpiSaúde Sociedade Científica, 7005-837 Évora, Portugal; 4 Faculdade de Ciências da Nutrição e Alimentação, Universidade do Porto, 4200-465 Porto, Portugal; 5 Programa Nacional para a Promoção da Alimentação Saudável, Direção-Geral da Saúde, 1049-005 Lisboa, Portugal; 6 Rheumatology Unit, Hospital dos Lusíadas, 1500-458 Lisboa, Portugal

**Keywords:** Mediterranean diet, socioeconomic, adults, southern Europe, Portugal, regional differences, age differences

## Abstract

The Mediterranean diet (MD) is recognized as one of the healthiest dietary patterns as it has been consistently associated with several beneficial health outcomes. Adherence to the MD pattern has been decreasing in southern European countries for the last decades, especially among low socioeconomic groups. The aim of this study was to assess the adherence to the MD in Portugal, to evaluate regional differences, and explore associated factors (sociodemographic, economic, and lifestyles behaviors). This study used the third data collection wave of the Epidemiology of Chronic Diseases Cohort Study (EpiDoC 3). MD adherence was assessed using the Portuguese-validated MD adherence score (MEDAS) questionnaire. Non-adjusted and adjusted logistic regression models were used to assess the risk factors for low MD adherence and individual MEDAS items. In this cross-sectional evaluation of the EpiDoC 3 cohort study (*n* = 5647), 28.8% of the Portuguese population had low adherence to a MD. Azores and Madeira had lower adherence to the MD than the rest of the country. Younger individuals in lower income categories (e.g., OR_finding it very difficult_ = 1.48; 95% CI 1.16–1.91) and with a lower educational level (e.g., OR_0–4 years_ = 2.63; 95% CI 2.09–3.32) had higher odds of having a lower adherence to the MD. Portuguese adults have a high prevalence of low adherence to the MD, especially among those who are younger and have lower socioeconomic status. Public health policies to promote adherence to the MD should pay special attention to these groups.

## 1. Introduction

Portugal has a high burden of diet-related chronic diseases [1] where more than 50% of the Portuguese adult population are overweight [2,3] and the prevalence of hypertension and diabetes reaches 36% and 10%, respectively [4]. According to the 2019 Global Burden of Disease Study, unhealthy dietary habits were the fifth risk factor (7.3%) contributing to the disability-adjusted life years in the Portuguese population [5]. Other diet-related risk factors such as high fasting plasma glucose, high blood pressure, high body-mass index (BMI), alcohol use, and high low-density lipoprotein cholesterol were the main risk factors contributing to healthy life years lost [5].

Portugal is a southern European country in the Mediterranean geocultural space with a traditional dietary pattern based on the Mediterranean diet (MD) principles, which was recognized by the United Nations Educational, Scientific and Cultural Organization as a humanity’s intangible cultural heritage [6,7,8].

The MD is characterized by a high consumption of fruit, vegetables, pulses, olive oil, and nuts and by a low consumption of meat and sweets [9]. Apart from these, the Portuguese MD has some particular aspects such as a high fish consumption and the widespread and frequent use of vegetable-based soups and stews [10]. These contribute to a higher intake of fiber, micronutrients and antioxidants, and monounsaturated and omega-3 fatty acids, all of which have, with a varying degree of evidence, been associated with health benefits [9]. The MD is recognized as a healthy dietary pattern and has been consistently associated with the prevention of several diseases and risk factors, namely cardiovascular disease [11,12,13,14,15,16,17,18], metabolic syndrome, adiposity, cognition, breast cancer, diabetes type 2, lipid profiles, inflammation, and blood pressure [16,19,20,21,22,23,24,25,26,27,28].

Data from previous studies, including data from the Portuguese Household Budget Survey, showed a decreasing trend in the adherence to the MD in the Portuguese population [8,29,30]. Additionally, other studies found that these downward trends were stronger in younger age groups and among those with the lowest incomes [31,32,33,34,35,36]. A compounding issue is that the adoption of Western-style processed foods high in salt, sugar, and fat is increasing and seems to be replacing the traditional MD [37].

There is an urgent need to counter this tendency and implement evidence-based strategies to recover the MD. In fact, the Portuguese government has created a working group to define and implement a set of measures to protect and increase the adherence to the MD. One of the objectives of this working group is to collect and provide data on the population adherence to the MD, as well as their associated factors.

Thus, the aims of our study were to: (a) assess the adherence to the MD in Portugal, using a nationally representative sample of the Portuguese population from the EpiDoC cohort, (b) to evaluate the regional differences in MD adherence, and (c) to explore associated factors (sociodemographic, economic, and lifestyles behaviors).

## 2. Materials and Methods

### 2.1. Study Design and Participants

**Figure 1:**
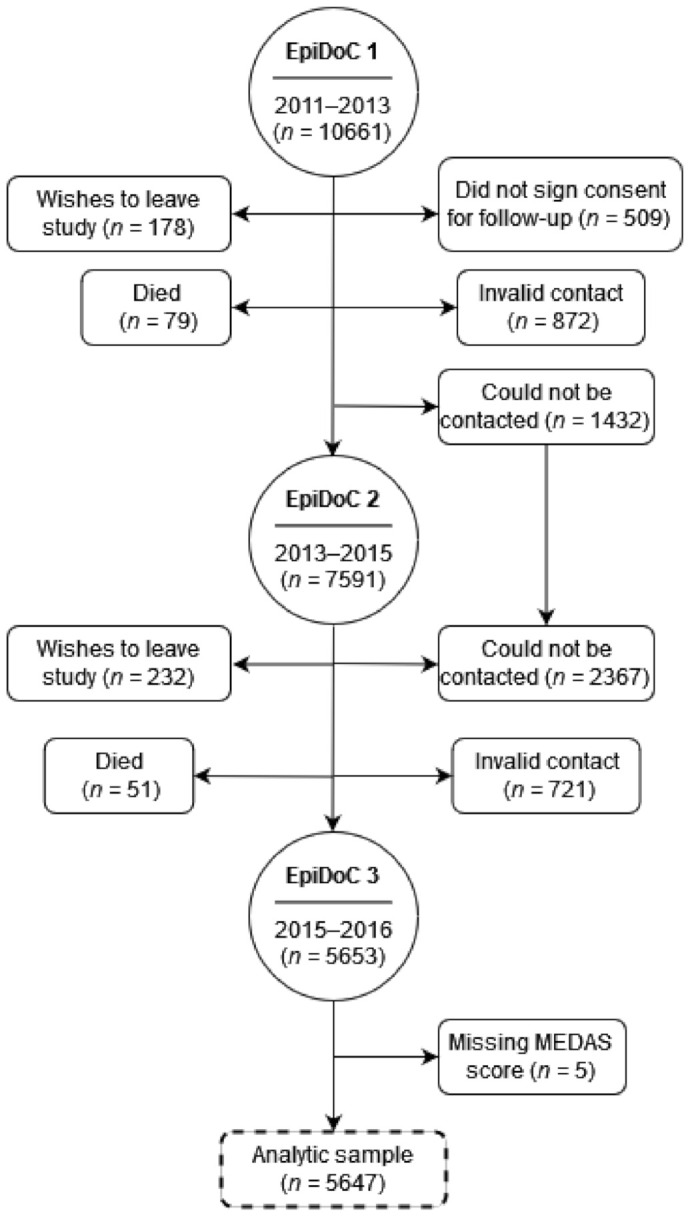
Flowchart of EpiDoC cohort. Participants that could not be contacted in EpiDoC 2 were not excluded from the study and the same contact was attempted at EpiDoC 3. Only data from EpiDoC 3 were used for the analytic sample.

The third follow-up evaluation wave of the Epidemiology of Chronic Diseases Cohort Study (EpiDoC 3) was used. The EpiDoC has been extensively described elsewhere [38]. Briefly, the EpiDoC is a cohort that aims to determine health determinants and health trajectories and their impact on health resource consumption in a nationally representative sample of Portuguese adults (18 years and older) living in the community and in private households in Portugal (on the mainland, and on the islands of Azores and Madeira) [38]. All of the 10,661 participants of the EpiDoC 1 study who signed the informed consent for follow-up and those who provided their telephone number were enrolled in the subsequent follow-up evaluations of the EpiDoC (EpiDoC 2, EpiDoC 3 and recently EpiDoC 4) [39]. The EpiDoC’s cohort flowchart is described in Figure 1, and the final analytic sample used in this manuscript was for 5647 participants.

The EpiDoC was performed according to the principles established by the Declaration of Helsinki and revised in 2013 in Fortaleza [40], as well as reviewed and approved by the National Committee for Data Protection and by the NOVA Medical School Ethics Committee [38].

### 2.2. Data Collection

Data were collected in the EpiDoC 3 from the 1st of September 2015 to the 28th of July 2016, using a structured questionnaire and delivered by a trained team of research assistants through computer-assisted telephone interviews. Database access was restricted to research team members and protected by a unique username and password. Portuguese-validated versions of the MD adherence score (MEDAS) [41], household food insecurity, [42] and EQ-5D-3L [43] were used, and the questionnaire was tested and improved to ensure comprehension of the questions and higher response rates.

### 2.3. Mediterranean Diet

MD adherence was assessed using the MEDAS questionnaire originally developed to assess compliance in the PREDIMED (PREvención con DIeta MEDiterránea) study [44]. This is a 14-item questionnaire with questions on dietary habits (e.g., olive oil usage) and daily or weekly frequency (depending on which item) of typical foods or food groups of the MD (e.g., olive oil, nuts, fruits, vegetables, pulses, seafood) and those that are not part of the traditional MD (e.g., red or processed meats, sweetened beverages and sweets, commercial bakery, or sugary desserts). The item was scored as a 1 if the MD adherence criteria was met or as a 0 otherwise. The final score ranged from 0 to 14, where a score ≤ 5 corresponds to a low adherence to the MD, a score between 6 and 9 corresponds to a moderate adherence to the MD, and a score ≥ 10 corresponds to high adherence to the MD [44]. This questionnaire was originally developed for the Spanish population and later validated for the Portuguese population [41].

### 2.4. Sociodemographic, Lifestyle and Health Variables

Information on sociodemographics (sex, years of education, and region) were collected at the baseline assessment (EpiDoC 1 study) and assumed constant throughout follow-up. Age was calculated as date of birth until the EpiDoC 3 interview. In the EpiDoC 3, the structured questionnaire included current employment status (employed full-time, part-time, unpaid household work, retired, unemployed, student, temporary incapacity to work), household income perception (able to live comfortably with present income, finding it difficult with present income, and finding it very difficult with present income), and the household food insecurity. To assess household food insecurity, a psychometric scale composed of 14 questions was adapted and validated for the Portuguese population from the Brazilian Food Insecurity Scale, which was originally adapted from the US Household Food Security Survey Module [42]. A score ranging from 0 to 14 was obtained from the total number of affirmative responses. According to this score, households were classified into four different categories of food insecurity: food security, low food insecurity, moderate food insecurity, and severe food insecurity [45,46].

Regarding health outcomes, the health-related quality of life was assessed using the European Quality of Life questionnaire with five dimensions and three levels (EQ-5D-3L) [47,48]. A higher EQ-5D score corresponds to a higher quality of life and ranges from −1 to 1. Healthcare resources consumption was also recorded, namely data regarding hospitalization in the previous 12 months (Yes/No), number of medical appointments, reduction of medical appointments due to economic difficulties (Yes/No), and reduction of medication due to economic difficulties (Yes/No).

Questions concerning lifestyle habits included frequency of intake of any type of alcohol (daily, occasionally, never), smoking habits (daily, occasionally, past smoker, never smoked), and frequency of physical activity (not regularly, regularly). Self-reported height and weight were collected, with BMI calculated as kg/m^2^ and categorized according to the World Health Organization classification [49].

### 2.5. Statistical Analyses

The sociodemographic, socioeconomic, and health characteristics of participants and non-participants of the EpiDoC 3 cohort were first compared to ensure the representativeness of the sample for the Portuguese population [36]. Weights for the Nomenclature of Territorial Unit for Statistics (NUTS II) region, sex, and age group were adjusted according to the comparison. Extrapolation weights for the EpiDoC 3 were computed and used in the subsequent statistical analyses. These were obtained by calibrating the extrapolation weights originally designed for the EpiDoC 1 study sample [38]. Absolute frequencies (*n*) and weighted proportions (%) were used to summarize categorical variables. Continuous variables were presented as weighted mean values and standard deviations (mean ± SD). The MD adherence prevalence was computed as weighted proportions according to NUTS II region, age group, sex, employment status, years of education, household income perception, BMI, and food insecurity. Participants were categorized into two groups—“low adherence to the MD” and “moderate or high adherence to the MD”—since there were not enough participants with high adherence to the MD. Non-adjusted logistic regression was used to assess the differences in lifestyles (smoking habits and physical activity), BMI, health-related characteristics (quality of life), and healthcare resource consumption between individuals with a low adherence to the MD and a moderate/high adherence to the MD.

Multivariable logistic regression models adjusted for age group, sex, educational level, employment status, and NUTS II were also used to further determine the possible differences between the MD adherence groups.

Non-adjusted and adjusted (for the same variables) logistic models were also used to determine the odds of MD adherence and individual MEDAS questionnaire items according to income perception categories. Estimates are presented as OR and the correspondent 95% CI. Significance level was set at 0.05. All analyses were performed using R (version 4.1.2) (R Foundation for Statistical Computing, Vienna, Austria).

## 3. Results

### 3.1. Adherence to the Mediterranean Diet in Portugal

**Figure 2:**
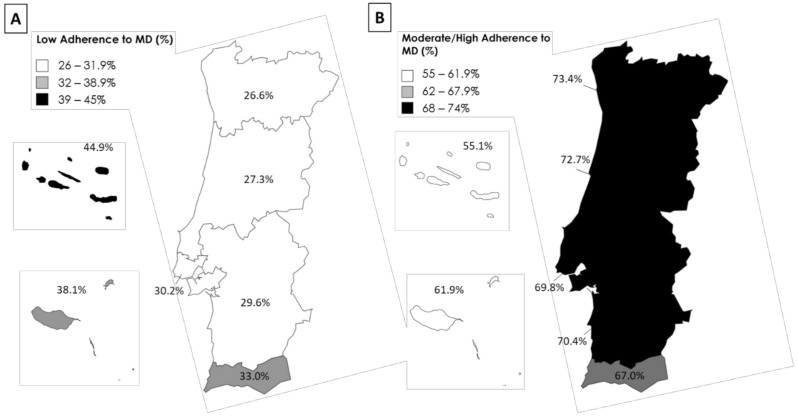
Low (**A**) and Moderate/High adherence (**B**) to the Mediterranean diet (MD) in Portugal according to the Nomenclature of territorial units for statistics II.

In the EpiDoC 3, a total of 5647 participants completed the MEDAS questionnaire. In this cohort study, 28.8% (*n* = 1545), 62.5% (*n* = 3630), and 8.7% (*n* = 472) reported a low, moderate and high adherence to the MD, respectively. Regional differences in a low and moderate/high adherence to the MD are shown in Figure 2. Azores had the highest percentage of a low adherence to the MD (44.9%) while the North had the highest percentage of a moderate/high adherence to the MD (73.4%).

### 3.2. Sociodemographic, Lifestyle and Health Characteristics Are Associated with a Low Adherence to the Mediterranean Diet in Portugal

Logistic regression models were performed to explore the risk factors associated with a low adherence to the MD. We found that women (OR = 0.75; 95% CI 0.66–0.86) and older adults (for example, participants in the 70–74 age group: OR = 0.29; 95% CI 0.19–0.43 vs. those 18–29 years) were less likely to have a low adherence to the MD, after adjusting for potential confounders. Individuals with 4 or less years of education were almost 3 times more at risk of having a low adherence to the MD (OR = 2.63; 95% CI 2.09–3.32) than individuals with 12 or more years of education.

Participants from the Azores (OR = 2.19; 95% CI 1.79–2.68) and Madeira islands (OR = 1.81; 95% CI 1.46–2.28) were almost twice as likely to have a lower adherence to the MD than the North of mainland Portugal.

Differences in adherence to the MD were also observed according to income perception. Individuals who found it difficult or very difficult to live with the present income were more likely to have a low adherence to the MD (OR = 1.42; 95% CI 1.16–1.74), (OR = 1.49; 95% CI 1.16–1.91). Similar trends were found when food insecurity was considered. Those living in households with moderate (OR = 1.72; 95% CI 1.31–2.24) and severe (OR = 2.57; 95% CI 1.58–3.23) food insecurity had a higher risk of having a low adherence to the MD.

Individuals who consumed alcohol daily (OR = 0.42; 95% CI 0.35–0.51) and had regular physical activity habits had lower odds of having a low adherence to the MD (OR = 0.53; 95% CI 0.46–0.60); on the other hand, those who smoked daily or occasionally had higher odds of having a low adherence to the MD (OR = 1.33; 95% CI 1.11–1.59).

Moreover, we observed that those with a higher quality of life score had lower odds of having a low adherence to the MD (OR = 0.48; 95% CI 0.38–0.59) (Table 1).

### 3.3. Low Income Is Associated with a Low Adherence to the Mediterranean Diet

Individual MEDAS items, stratified by income perception categories, are shown in Table 2, and the odds of scoring 1 on those individual items according to perception income are shown in Table 3. Individuals in the lower income categories (finding it difficult or very difficult to live with present income) were less likely to score 1 for individual items from the MD adherence questionnaire than those who were able to live comfortably with their present income. For example, participants who were finding it more difficult to live with their present income were less likely to consume at least 4 tablespoons of olive oil per day (OR_finding it difficult_ = 0.75; 95% CI 0.60–0.93 and OR_finding it very difficult_ = 0.65; 95% CI 0.48–0.86), to consume 2 or more servings of vegetables per day (OR_finding it difficult_ = 0.74; 95% CI 0.60–0.91 and OR_finding it very difficult_ = 0.82; 95% CI 0.64–1.07), to consume 3 or more servings of fish/seafood per week (OR_finding it difficult_ = 0.65; 95% CI 0.54–0.78 and OR_finding it very difficult_ = 0.49; 95% CI 0.39–0.61), and to consume 3 or more servings of nuts per week (OR_finding it difficult_ = 0.58; 95% CI 0.45–0.78 and OR_finding it very difficult_ = 0.49; 95% CI 0.34–0.71), after adjusting for sex, age group, educational level, employment status, and NUTS II (Table 3).

## 4. Discussion

This study showed that a high percentage of community-dwelling Portuguese adults had a low adherence to the MD. Participants with fewer years spent in formal education and with a lower income were more likely to have a low adherence to the MD.

We found that 29%, 63% and 9% of Portuguese adults ≥ 18 years had a low, moderate and high adherence to the MD, respectively. In a study with Portuguese university students, only 12.5% had a high adherence to the MD during 2017–18 [50]. Furthermore, a 2020 Directorate-General of Health report stated that 26% of 1000 participants ≥ 16 years had a high adherence to the MD [51]. Such an increase from 9% to 26% over the course of 4–5 years is very promising.

However, in general, adherence to the MD pattern has been decreasing in southern European countries for the last decades [33,52,53,54,55]. Other studies have shown that this downward trend was more evident in those with a lower socioeconomic status [31,32,34,35,36]. In line with others, participants in our study who found it harder to live with their present income were more likely to have a low adherence to the MD. Higher costs of healthier foods, typically belonging to a MD pattern, have been suggested to be an important factor for the downward trend in MD adherence [56]. Spanish and British studies found that a greater adherence to the MD was associated with higher food costs [57,58,59]. The authors stated that the potential economic barriers might be offset by a reduction in healthcare costs from the reduction of unhealthy food consumption and subsequent long-term non-communicable diseases that may develop [59]. In fact, the very robust association between health inequalities and socioeconomic status can be partially explained by differences in dietary intake [60,61,62,63,64]. Moreover, a study on Portuguese adults showed that, regardless of sex, those with a higher income and higher education were more likely to consume healthier foods (i.e., fish, fruit, and vegetables) [65]. This is in line with our results, as individuals in the lower income categories were less likely to have a high consumption of fruit and fish/seafood, among other foods characteristic of the MD. As others have suggested, public health policies aiming to promote healthy dietary habits should pay special attention to socially vulnerable groups [65].

The economic crisis, starting around 2007, accelerated the decline in MD adherence in southern European countries, as observed by an Italian study where higher socioeconomic status was strongly associated with a higher adherence to the MD in 2007–2010, whereas no association was detected in the years prior [33]. Future studies regarding a more recent period may clarify if the association between MD adherence and socioeconomic status persisted following the economic crisis in Portugal.

In the EpiDoC 3, older participants were more likely to have a greater adherence to the MD than younger ones, even in analyses adjusted for socioeconomic status. These findings may indicate that younger generations are not adopting the MD as much as older generations did.

Furthermore, our results showed that a low adherence to the MD was significantly and independently associated with a lower score for quality of life, which is in line with previously reported findings [66,67]. A lower quality of life is likely to be a partial cause and a partial consequence of a lower adherence to the MD. However, and despite the low adherence to the MD in Portugal and elsewhere, targeted nutrition public health policies have showed promise. For example, the Portuguese National Program for the Promotion of Healthy Eating started in 2012 and aimed to, through a series of concerted actions, promote healthy eating habits to the Portuguese population. In a short period, the program has already seen the reduction of sodium in bread [68], the taxation of sugar-sweetened beverages [69], and the change in the availability of unhealthy foods at schools and public spaces [70]. The Portuguese Ministry of Agriculture, Forestry and Rural Development is also actively promoting the MD by supporting small farmers and promoting the production of locally sourced foods [71]. The MD has not only been associated with numerous health benefits, but it is also considered a sustainable diet with fewer environmental impacts than a typical western diet [72]. An increase in the adherence to the MD in the Portuguese population would have important beneficial effects on diet-related risk factors, which could eventually lead to an increase in the disability-free life expectancy. This study has some limitations. Adherence to the MD, measured by the MEDAS questionnaire, was only collected at the EpiDoC 3 (2015–2016) and not at previous data collection waves. This had two implications: (a) we could not observe the evolution of MD adherence over time; and (b) the analysis was cross-sectional. However, reverse causality may not be a problem for every risk factor as most precede and cannot be changed by MD adherence (age, sex, NUTSII, education, and socioeconomic status). That being said, we cannot exclude whether MD adherence increased quality of life or quality of life increased MD adherence, assuming that all confounders have been accounted for. Most data were self-reported and collected during a telephone interview, which increases the risk for misclassification. For example, MEDAS questions may be subject to recall bias and socially desirability bias, which could lead to a sub-estimation of the low MD adherence in the EpiDoC 3. Moreover, income was assessed as the difficulty to live with the present income, which may reflect other psychological constructs and not the true income level. Finally, years spent in formal education was only collected at the EpiDoC 1 and was, therefore, assumed constant throughout follow-up. However, since the youngest age category is 18–29 and the highest education category is 12 or more years spent in formal education, it is unlikely that after 4 years the education classification would have changed considerably.

The study strengths include an established methodology, a nationally representative community-based sample (with age and sex strata distribution according to the Portuguese population), and a well-trained research assistant team that administered the questionnaire. Moreover, the MD adherence was assessed using the MEDAS questionnaire validated for the Portuguese population and potential confounders were included in the logistic regression analysis.

## 5. Conclusions

Almost one third of Portuguese adults reported having a low adherence to the MD. Lower socioeconomic status and younger age were consistently associated with a lower adherence to the MD. Future public health policies in Portugal aimed at increasing the adherence to the MD should target younger and socially vulnerable groups.

## Figures and Tables

**Table 1:** Sociodemographic, socioeconomic, lifestyles and health characteristics of individuals with low and moderate/high adherence to the Mediterranean diet (MD), and logistic regression models for the odds of having low adherence vs. moderate/high adherence to the MD.

	All (*n* = 5647)	Low Adherence to MD (*n* = 1545)	Moderate/High Adherence to MD (*n* = 4102)	Non-Adjusted OR (95% CI)	Adjusted OR (95% CI) *
***Socioeconomic and demographic***					
***Sex, n (%)***					
*Women*	3607 (52.5)	921 (45.6)	2686 (55.3)	**0.78 (0.69–0.88)**	**0.75 (0.66–0.86)**
***Age, years, n (%)***					
*18–29*	355 (15.4)	152 (19.7)	203 (13.6)	1 (ref.)	1 (ref.)
*30–39*	605 (19.1)	204 (25.0)	401 (16.7)	**0.68 (0.52–0.89)**	**0.64 (0.48–0.85)**
*40–49*	1049 (18.3)	295 (17.4)	754 (18.6)	**0.52 (0.41–0.67)**	**0.44 (0.33–0.57)**
*50–59*	1143 (15.9)	283 (13.1)	860 (17.0)	**0.44 (0.34–0.56)**	**0.33 (0.25–0.43)**
*60–69*	1112 (13.7)	241 (10.9)	871 (14.9)	**0.37 (0.29–0.48)**	**0.29 (0.21–0.40)**
*70–74*	491 (6.7)	107 (3.3)	384 (8.1)	**0.37 (0.28–0.50)**	**0.29 (0.19–0.43)**
*≥75*	892 (10.9)	263 (10.6)	629 (11.1)	**0.56 (0.43–0.72)**	**0.45 (0.31–0.65)**
***Education, years, n (%)***					
*0–4*	2203 (27.4)	607 (24.6)	1596 (28.5)	**1.61 (1.34–1.94)**	**2.63 (2.09–3.32)**
*5–9*	1335 (23.1)	410 (28.4)	925 (20.9)	**1.87 (1.54–2.29)**	**2.41 (1.95–2.99)**
*10–12*	1128 (27.7)	338 (30.2)	790 (26.7)	**1.81 (1.48–2.22)**	**1.78 (1.44–2.20)**
*>12*	967 (21.8)	185 (16.8)	782 (23.8)	1 (ref.)	1 (ref.)
***NUTS II, n (%)***					
*North*	1659 (36.5)	380 (33.7)	1279 (37.6)	1 (ref.)	1 (ref.)
*Center*	1086 (23.2)	268 (22.0)	818 (23.7)	1.10 (0.92–1.32)	1.15 (0.95–1.38)
*Lisbon*	1131 (24.8)	303 (26.0)	828 (24.3)	**1.23 (1.03–1.47)**	**1.45 (1.21–1.75)**
*Alentejo*	320 (7.2)	87 (7.4)	233 (7.1)	1.26 (0.95–1.64)	1.40 (1.06–1.85)
*Algarve*	183 (3.7)	56 (4.3)	127 (3.5)	**1.48 (1.06–2.06)**	**1.59 (1.11–2.24)**
*Azores*	657 (2.2)	254 (3.4)	403 (1.7)	**2.12 (1.75–2.58)**	**2.19 (1.79–2.68)**
*Madeira*	611 (2.4)	197 (3.2)	414 (2.1)	**1.60 (1.30–1.96)**	**1.81 (1.46–2.24)**
***Employment status, n (%)***					
*Employed full/part-time*	2444 (53.0)	700 (57.2)	1744 (51.4)	1 (ref.)	1 (ref.)
*Unpaid household worker*	450 (4.7)	116 (4.9)	334 (4.7)	0.87 (0.69–1.08)	0.83 (0.63–1.09)
*Retired*	2007 (26.4)	472 (18.4)	1535 (29.6)	**0.77 (0.67–0.88)**	**0.75 (0.59–0.95)**
*Unemployed/Student/Temporary* *incapacity to work*	706 (15.9)	226 (19.5)	480 (14.4)	1.17 (0.98–1.40)	1.03 (0.85–1.25)
***Income perception, n (%)***					
*Able to live comfortably with present income*	1027 (22.8)	238 (21.6)	789 (23.2)	1 (ref.)	1 (ref.)
*Able to live with present income*	2293 (43.2)	583 (42.4)	1710 (43.5)	1.13 (0.95–1.34)	1.13 (0.94–1.37)
*Finding it difficult with present income*	1565 (25.0)	459 (25.8)	1106 (24.6)	**1.38 (1.15–1.65)**	**1.42 (1.16–1.74)**
*Finding it very difficult with present income*	675 (9.1)	201 (10.2)	474 (8.6)	**1.41 (1.13–1.75)**	**1.48 (1.16–1.91)**
***Food security status, n (%)***					
*Food security*	4151 (80.7)	1024 (77.9)	3127 (81.8)	1 (ref.)	1 (ref.)
*Low food insecurity*	959 (14.1)	274 (19.0)	676 (13.8)	**1.24 (1.06–1.45)**	**1.18 (1.00–1.40)**
*Moderate food insecurity*	286 (3.5)	101 (6.6)	185 (3.1)	**1.67 (1.29–2.14)**	**1.72 (1.31–2.24)**
*Severe food insecurity*	144 (1.8)	61(4.5)	83 (1.4)	**2.24 (1.60–3.14)**	**2.27 (1.58–3.23)**
***Lifestyle***					
***Body Mass Index, kg/m^2^, n (%)***					
*Underweight*	88 (2.1)	27 (2.9)	61 (1.8)	1.25 (0.78–1.97)	1.25 (0.76–2.01)
*Normal weight*	2009 (44.5)	525 (46.1)	1484 (43.9)	1 (ref.)	1 (ref.)
*Overweight*	2098 (37.7)	504 (34.6)	1594 (38.8)	0.89 (0.78–1.03)	0.92 (0.79–1.07)
*Obesity*	979 (15.7)	280 (16.3)	699 (15.5)	1.13 (0.95–1.34)	**1.22 (1.01–1.46)**
***Alcohol intake, n (%)***					
*Never*	1945 (30.6)	577 (31.3)	1368 (30.3)	1 (ref.)	1 (ref.)
*Daily*	1565 (29.7)	268 (19.0)	1297 (33.9)	**0.49 (0.42–0.58)**	**0.42 (0.35–0.51)**
*Occasionally*	2020 (39.6)	616 (49.7)	1404 (35.7)	1.04 (0.91–1.19)	0.97 (0.84–1.13)
***Smoking habits, n (%)***					
*Never smoked*	3150 (57.3)	883 (54.4)	2627 (58.5)	1 (ref.)	1 (ref.)
*Past smoker*	1149 (21.1)	276 (18.2)	873 (22.3)	0.94 (0.80–1.10)	0.91 (0.77–1.09)
*Smokes daily/occasionally*	876 (21.5)	300 (27.4)	576 (19.3)	**1.55 (1.32–1.82)**	**1.33 (1.11–1.59)**
***Physical activity, n (%)***					
*Regularly*	2370 (44.2)	474 (35)	1896 (47.9)	**0.52 (0.45–0.58)**	**0.53 (0.46–0.60)**
***Quality of life***					
*EQ-5D-3L, mean (±SD)*	0.7 (0.3)	0.8 (±0.3)	0.8 (±0.3)	**0.56 (0.47–0.67)**	**0.48 (0.38–0.59)**
***Healthcare resources consumption***					
*Was hospitalized since last contact, n (%)*	737 (11.0)	195 (9.9)	542 (11.5)	0.95 (0.80–1.13)	0.99 (0.82–1.19)
*Went to medical appointments in hospitals since last contact, n (%)*	2043 (43.1)	532 (37.0)	1511 (45.5)	0.94 (0.82–1.07)	0.94 (0.82–1.08)
*Went to medical appointments in primary health care centers since last contact, n (%)*	4126 (91.9)	1073 (90.5)	3053 (92.5)	**0.71 (0.57–0.88)**	0.82 (0.65–1.05)
*Number of medical appointments in public sector since last contact, mean (±SD)*	3.57 (4.1)	3.2 (±3.4)	3.7 (±4.3)	**0.98 (0.96–0.99)**	0.98 (0.97–1.00)
*Number of medical appointments in private sector since last contact, mean (±SD)*	1.35 (3.4)	1.2 (±4.0)	1.4 (±3.1)	**0.96 (0.94–0.98)**	0.98 (0.95–1.00)
***Chronic disease management***					
*Reduction in medication due to economic constraints, n (%)*	496 (6.0)	156 (6.0)	340 (6.1)	**1.25 (1.02–1.52)**	**1.30 (1.06–1.61)**
*Reduction in medical appointments due to economic constraints, n (%)*	516 (6.4)	146 (6.1)	370 (6.5)	1.05 (0.86–1.29)	1.03 (0.83–1.26)
**MEDAS score, mean (±SD)**	6.64 (2.1)	4.1 (1.0)	7.7 (1.5)	-	-

* Adjusted for sex, age group, educational level, employment status and Nomenclature of territorial units for statistics II (NUTS II). Values are displayed as *n* (%) or mean (±SD). Participants with moderate/high adherence to MD were used as the reference. Estimates with a significance level < 0.05 have been highlighted in bold. EQ-5D-3L, European Quality of Life questionnaire with five dimensions and three levels; MD, Mediterranean diet; MEDAS, MD adherence score; ref., reference.

**Table 2:** Individual MEDAS items according to four income perception categories.

	All (*n* = 5561)	Able to Live Comfortably with Present Income (*n* = 1027)	Able to Live with Present Income (*n* = 2293)	Finding It Difficult with Present Income (*n* = 1566)	Finding It Very Difficult with Present Income (*n* = 675)
***Individual MEDAS items, n (%)***					
*Olive oil as principal source of fat for cooking*	4897 (87.4)	925 (87.7)	2051 (89.8)	1341 (83.6)	580 (85.6)
*Olive oil (>4 Tbsp per day)*	1109 (21.9)	258 (26.5)	501 (23.0)	259 (17.1)	91 (18.2)
*Vegetables (≥2 servings per day)*	1266 (21.4)	274 (22.9)	537 (22.1)	311 (18.8)	144 (21.9)
*Fruit (≥3 units per day)*	1770 (29.7)	385 (30.6)	825 (32.4)	415 (27.5)	145 (20.8)
*Red meat, hamburgers or sausages (<1 serving per day)*	4131 (67.7)	730 (66.1)	1677 (65.9)	1199 (71.3)	525 (70.1)
*Butter, margarine or cream (<1 per day)*	3851 (68.4)	693 (70.2)	1553 (66.9)	1105 (68.6)	500 (70.1)
*Carbonated and/or sugar-sweetened beverages (<1 per day)*	4745 (82.6)	863 (79.0)	1966 (83.4)	1334 (84.1)	582 (83.5)
*Wine (≥7 glasses per week)*	1250 (23.6)	209 (20.1)	561 (23.9)	347 (25.7)	133 (25.5)
*Pulses (≥3 servings per week)*	1340 (24.5)	295 (24.5)	575 (23.2)	335 (20.8)	135 (22.3)
*Fish/seafood (≥3 servings per week)*	3106 (57.7)	674 (66.3)	1334 (58.3)	803 (53.9)	295 (44.1)
*Commercial (not homemade) pastries (<2 servings per week)*	3494 (59.9)	576 (54.2)	1403 (60.6)	1015 (60.2)	500 (69.4)
*Nuts (≥3 servings per week)*	753 (15.1)	246 (25.1)	313 (14.1)	149 (11.3)	45 (5.4)
*Preference to consume chicken, turkey or rabbit meat instead of beef, pork, hamburger or sausages*	3442 (61.1)	652 (61.0)	1412 (60.5)	972 (62.9)	406 (59.5)
*Boiled vegetables, pasta, rice or other dishes with a sauce of tomato, garlic, onion or leeks sautéed in olive oil (≥2 times per week)*	2341 (47.6)	499 (53.9)	982 (48.9)	609 (41.7)	251 (41.9)

All values are displayed as *n* (%). One tablespoon is equal to 14–15 mL and one glass is equal to 150 mL. One serving of vegetables is equal to 200 g, one serving of red meat, hamburger and sausages is 100–150 g, one serving of butter, margarine or cream is equal to 12 g, one serving of legumes is 150 g, one serving of fish is 100–150 g and one serving of seafood is 200 g, and one serving of nuts is equal to 30 g; MD, Mediterranean diet; MEDAS, MD adherence score; Tbsp, tablespoon.

**Table 3:** Odds of individual MEDAS items according to income perception categories.

	Able to Live with Present Income (*n* = 2293)		Finding It Difficult with Present Income (*n* = 1566)		Finding It Very Difficult with Present Income (*n* = 675)	
	Non-Adjusted OR (95% CI)	Adjusted OR (95% CI) *	Non-adjusted OR (95% CI)	Adjusted OR (95% CI) *	Non-Adjusted OR (95% CI)	Adjusted OR (95% CI) *
***Individual MEDAS items***						
*Olive oil as principal source of fat for cooking*	0.96 (0.75–1.22)	1.01 (0.78–1.32)	**0.68 (0.52–0.86)**	**0.73 (0.55–0.96)**	**0.70 (0.51–0.94)**	0.79 (0.56–1.11)
*Olive oil (>4 Tbsp per day)*	**0.84 (0.70–0.99)**	0.94 (0.79–1.13)	**0.59 (0.49–0.72)**	**0.75 (0.60–0.93)**	**0.46 (0.36–0.60)**	**0.65 (0.48–0.86)**
*Vegetables (≥2 servings per day)*	**0.84 (0.71–0.99)**	0.88 (0.74–1.06)	**0.68 (0.57–0.82)**	**0.74 (0.60–0.91)**	**0.74 (0.59–0.94)**	0.82 (0.64–1.07)
*Fruit (≥3 units per day)*	0.94 (0.80–1.09)	0.90 (0.76–1.06)	**0.60 (0.51–0.71)**	**0.55 (0.46–0.67)**	**0.45 (0.36–0.57)**	**0.41 (0.32–0.52)**
*Red meat, hamburgers or sausages (<1 serving per day)*	1.11 (0.94–1.30)	1.05 (0.88–1.25)	**1.34 (1.12–1.61)**	1.12 (0.91–1.36)	**1.41 (1.13–1.78)**	1.03 (0.80–1.33)
*Butter, margarine or cream (<1 per day)*	1.01 (0.86–1.18)	0.98 (0.83–1.16)	1.18 (0.99–140)	1.09 (0.90–1.32)	**1.37 (1.10–1.70)**	1.22 (0.96–1.55)
*Carbonated and/or sugar-sweetened beverages (<1 per day)*	1.14 (0.92–1.39)	1.10 (0.88–1.37)	1.11 (0.89–1.38)	0.97 (0.76–1.24)	1.17 (0.89–1.55)	0.92 (0.67–1.26)
*Wine (≥7 glasses per week)*	**1.27 (1.06–1.52)**	0.95 (0.77–1.18)	1.12 (0.92–1.36)	0.84 (0.66–1.07)	0.96 (0.75–1.22)	**0.72 (0.53–0.97)**
*Pulses (≥3 servings per week)*	**0.83 (0.70–0.98)**	0.88 (0.74–1.05)	**0.68 (0.57–0.81)**	**0.73 (0.60–0.89)**	**0.62 (0.49–0.78)**	**0.66 (0.51–0.85)**
*Fish/seafood (≥3 servings per week)*	**0.72 (0.62–0.84)**	**0.81 (0.69–0.96)**	**0.55 (0.47–0.65)**	**0.65 (0.54–0.78)**	**0.40 (0.33–0.49)**	**0.49 (0.39–0.61)**
*Commercial (not homemade) pastries (<2 servings per week)*	**1.23 (1.06–1.43)**	1.03 (0.88–1.21)	**1.46 (1.24–1.71)**	1.09 (0.91–1.30)	**2.22 (1.80–2.74)**	**1.51 (1.19–1.91)**
*Nuts (≥3 servings per week)*	**0.50 (0.42–0.60)**	**0.69 (0.57–0.85)**	**0.33 (0.27–0.42)**	**0.58 (0.45–0.74)**	**0.23 (0.16–0.31)**	**0.49 (0.34–0.71)**
*Preference to consume chicken, turkey or rabbit meat instead of beef, pork, hamburger or sausages*	0.91 (0.78–1.06)	0.97 (0.82–1.14)	0.94 (0.80–1.11)	0.96 (0.80–1.15)	0.84 (0.69–1.03)	0.82 (0.67–1.03)
*Boiled vegetables, pasta, rice or other dishes with a sauce of tomato, garlic, onion or leeks sautéed in olive oil (≥2 times per week)*	**0.79 (0.68–0.91)**	0.92 (0.79–1.08)	**0.68 (0.58–0.79)**	0.87 (0.73–1.04)	**0.62 (0.51–0.76)**	0.87 (0.70–1.09)

* Adjusted for sex, age group, educational level, employment status and Nomenclature of territorial units for statistics II (NUTS II). “Able to live comfortably with present income” was the reference category (*n* = 1027) for the four income perception categories (exposure). Participants with criteria for 0 points in individual MEDAS questionnaire items (outcome) were used as the reference. One tablespoon is equal to 14–15 mL and one glass is equal to 150 mL. One serving of vegetables is equal to 200 g, one serving of red meat, hamburger and sausages is 100–150 g, one serving of butter, margarine or cream is equal to 12 g, one serving of legumes is 150 g, one serving of fish is 100–150 g and one serving of seafood is 200 g, and one serving of nuts is equal to 30 g. Estimates with a significance level < 0.05 have been highlighted in bold. MD, Mediterranean diet; MEDAS, MD adherence score; Tbsp, tablespoon.

## Data Availability

The datasets, codebook and analytic code are available pending application and approval by the EpiDoC steering committee (nms.unl.pt/pt-pt/Investigação/Grupos-de-Investigação/Detalhe/investigationgroupid/2062, accessed on 31 January 2022).
